# Recent insights into the genotype–phenotype relationship from massively parallel genetic assays

**DOI:** 10.1111/eva.12846

**Published:** 2019-08-11

**Authors:** Harry Kemble, Philippe Nghe, Olivier Tenaillon

**Affiliations:** ^1^ Infection, Antimicrobials, Modelling, Evolution, INSERM, Unité Mixte de Recherche 1137 Université Paris Diderot, Université Paris Nord Paris France; ^2^ École Supérieure de Physique et de Chimie Industrielles de la Ville de Paris (ESPCI Paris), UMR CNRS‐ESPCI CBI 8231 PSL Research University Paris Cedex 05 France

**Keywords:** distribution of fitness effects, epistasis, fitness landscapes, genotype–phenotype maps, high‐throughput genetics, phenotypic models

## Abstract

With the molecular revolution in Biology, a mechanistic understanding of the genotype–phenotype relationship became possible. Recently, advances in DNA synthesis and sequencing have enabled the development of deep mutational scanning assays, capable of scoring comprehensive libraries of genotypes for fitness and a variety of phenotypes in massively parallel fashion. The resulting empirical genotype–fitness maps pave the way to predictive models, potentially accelerating our ability to anticipate the behaviour of pathogen and cancerous cell populations from sequencing data. Besides from cellular fitness, phenotypes of direct application in industry (*e.g*. enzyme activity) and medicine (*e.g*. antibody binding) can be quantified and even selected directly by these assays. This review discusses the technological basis of and recent developments in massively parallel genetics, along with the trends it is uncovering in the genotype–phenotype relationship (distribution of mutation effects, epistasis), their possible mechanistic bases and future directions for advancing towards the goal of predictive genetics.

## 1 INTRODUCTION

The relationship between genotype, understood in the age of molecular biology as nucleic acid or polypeptide sequence, and phenotype, any trait from the molecular to organismal scale, is of profound importance across biological disciplines. Direct genotype–phenotype mapping has recently become possible on a large scale thanks primarily to the creative use of next‐generation sequencing (NGS) technologies to develop massively parallel assays capable of measuring a particular phenotype for each genetic variant in a large mixed library.

One of the most amenable phenotypes to measurement by such assays is microbial or cell fitness, which simply requires the tracking of genotype frequencies during laboratory propagation of a mixed culture (Hietpas, Jensen, & Bolon, 2011). The increasing availability of the resulting genotype–fitness maps opens the door to predictive models, potentially accelerating our ability to anticipate the behaviour of pathogens and cancerous cells from sequencing data. The quantification of other phenotypes alongside fitness, such as expression level or enzymatic activity, can enhance genotype–fitness models by providing mechanistic descriptions of fitness and intermediate phenotypes, themselves of interest to bioengineering (*e.g*. rational design of high‐performance enzymes) and precision medicine (Lehner, 2013). Further, the assays presented in the following section can be used directly for the selection of industrially or medically useful phenotypes, as exemplified by the 2018 Nobel Prizes in Chemistry for antibody and enzyme engineering.

This review synthesizes technological developments in massively parallel genetics, the trends it is uncovering in the genotype–phenotype relationship and their potential mechanistic bases. The three main sections review mutation effects in, respectively, single genes, two or a few genes, and entire genomes, with each section discussing the distributions of mutational effects (DME) and the interaction between mutations (epistasis).

## 2 ONE GENE

### 2.1 Experimental history of large‐scale genotype–phenotype mapping for single genes

Empirical snapshots of the local, or occasionally global, genotype–phenotype map for short sequences have recently become possible through combinations of high‐throughput technologies. The technical challenge may be broken down as follows: (a) the number of possible nucleic acid or protein sequence variants grows exponentially with sequence length; (b) to draw statistical conclusions about the genotype–phenotype relationship for even very short sequences (*e.g*.>5‐mer), a large number (>10^3^) of sequence variants must be characterized; (c) currently, the only economical way of generating large numbers of sequence variants is in bulk; and (d) to characterize a phenotype of a large number of pooled sequence variants, not only do we need a high‐throughput phenotyping technology, but also a high‐throughput way to trace phenotypes back to their sequence.

The earliest strategy used to achieve this last challenge was *phage display* (Scott & Smith, 1990; Smith, 1985). It allows biochemical phenotypes of polypeptides, such as binding affinity, to be easily traced back to their coding DNA sequence by fusing the polypeptide of interest to a viral coat protein and thus making it accessible for biochemical analysis while remaining physically associated with the DNA from which it is expressed. Soon after the invention of phage display, alternative display systems were developed (bacteria, yeast, ribosome, mRNA) (Levin & Weiss, 2006), but it was not until about 20 years after that a display strategy was used to attempt comprehensive local genotype–phenotype characterization (Pál, Kouadio, Artis, Kossiakoff, & Sidhu, 2006).

In this study, single point mutations were systematically introduced into the 35‐aa receptor‐binding site of human growth hormone using Kunkel mutagenesis (Kunkel, Bebenek, & McClary, 1991), a phage display library was constructed, the library was screened for binding to either a structure‐specific antibody or the human growth hormone receptor (to assess structural stability and receptor‐binding affinity, respectively), and a sample of screened clones was Sanger‐sequenced to achieve quasi‐quantitative measures for these two phenotypes. This first comprehensive *deep mutational scanning* study produced several novel insights: (a) the native protein fold was highly tolerant to mutations in the targeted solvent‐exposed positions (with the exceptions of cysteine and proline), with all positions showing a similar level of robustness; (b) hydrophobic residues were generally more stabilizing than hydrophilic ones, a very surprising result for a solvent‐exposed region; (c) at the majority of positions, mutations existed that resulted in both greater stability and stronger receptor binding than the wild type; (d) binding affinity was less robust to mutation than stability, with robustness being position‐specific; (e) binding affinity robustness did not relate strongly to sequence conservation across species; and (f) physicochemical similarity of residue side chains did not correlate strongly with phenotypic effects (Pál et al., 2006).

Although hugely informative, only a few studies employing this methodological strategy have been performed, as it still involves one low‐throughput step—Sanger sequencing. Microarray binding assays provide a higher‐throughput approach for the specific case of assessing binding of a predefined array of short nucleic acid sequences to a protein (Badis et al., 2009), but ultimately it was the arrival of massively parallel sequencing technologies that allowed large‐scale genotype–phenotype mapping studies to flourish. Similarly to the aforementioned Sanger‐sequencing study, the earliest such study used phage display of human WW domain variants coupled with (weak) selection for binding to its peptide ligand, with Illumina sequencing of pre‐ and postselection libraries to again give a quasi‐quantitative measure of binding affinity (Fowler et al., 2010). The conclusions differed substantially: 97% of the library variants bound the ligand less tightly than did the wild type; mutational intolerance of the different positions correlated strongly with evolutionary conservation; the core ligand binding region was generally intolerant to mutation; each position appeared to possess a unique mutational preference spectrum; and global thermodynamic stability was concluded to be the primary determinant of binding affinity. Some of the inconsistencies between these two studies may have a technical basis, but they also provide a first hint that the genotype–phenotype relationship, even for a given biochemical phenotype, may vary substantially between proteins.

**Figure 1:**
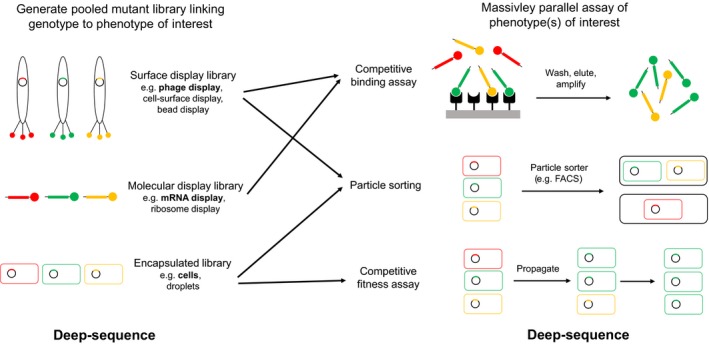
Mutant library types and massively parallel sequencing‐resolved assays for high‐throughput genotype–phenotype mapping. Bulk mutagenesis is used to construct an in vitro or in vivo genotype library, with the phenotype(s) of interest associated with the genotype either by “display” or encapsulation. Phenotypic measurements are then linked to genotypes by deep‐sequencing of the library before and after some selection procedure. Selection procedures include binding to a target for direct binding phenotypes, particle sorting for any optically assayable phenotype (*e.g*. fluorescence or cell dimensions) and simple propagation under selective conditions for competitive fitness. Filled rectangles (straight or curved): nucleic acids; filled circles: proteins. Illustrated library examples are named in bold

With the advent of high‐throughput sequencing, the limitation becomes the coupling of phenotype measurement with sequencing. Creative approaches have been developed based on surface display to assess a mutant protein library for binding to other proteins, DNA, RNA and small molecule ligands, as well as amenable enzymatic activities such as ubiquitination (Fowler & Fields, 2014; Starita et al., 2013). Further, particle sorting techniques like cell and microfluidic‐droplet sorting can be used to place variants into phenotypic (*e.g*. fluorescence, cell size) bins which are then each subjected to deep‐sequencing (Fowler & Fields, 2014; Kinney, Murugan, Callan, & Cox, 2010; McLaughlin, Poelwijk, Raman, Gosal, & Ranganathan, 2012; Noderer et al., 2014; Sarkisyan et al., 2016; Whitehead et al., 2012) (Figure 1).

To overcome this limitation, in vitro fluorescence‐coupled microarray and flow‐cell techniques have been developed to *directly* measure the biochemical phenotypes of a large number of nucleic acid or protein mutants in parallel, and associate these measurements with the respective genotypes (Boyle et al., 2017; Buenrostro et al., 2014; Geertz, Shore, & Maerkl, 2012; Guenther et al., 2013; Layton, McMahon, & Greenleaf, 2019; Maerkl & Quake, 2009; Nutiu et al., 2011; Shultzaberger, Maerkl, Kirsch, & Eisen, 2012). The current limit of such methods is their requirement for highly specialized equipment and expertise, restricting their use to just a handful of laboratories.

One particular phenotype has proven generally amenable to easy and affordable experimental genotype–phenotype coupling, however. It allows precise, quantitative estimates and is of direct evolutionary significance: competitive cellular fitness (Hietpas et al., 2011) (Figure 1). In the original EMPIRIC (*Extremely Methodical and Parallel Investigation of Randomized Individual Codons*) experiment, a 9‐a.a. region of *S. cerevisiae* Hsp90 (an essential and highly conserved eukaryotic chaperone protein) was comprehensively mutagenized to create a library of all possible single point mutants. The fitness impact of all mutations was then directly measured in bulk, by serially propagating the library in conditions where expression of the native *hsp90* copy was repressed, and using deep‐sequencing to sample mutant frequencies at several time points over a few days. Because the wild‐type sequence was included in the library and the wild‐type generation time was known, the competitive fitness of each mutant could be estimated relative to the wild type as a selection coefficient, by taking the change in the ratio of mutant to wild‐type reads as a function of wild‐type generation time.

This data set provided an important empirical insight: the distribution of fitness effects in this individual gene was bimodal, with a fairly equal proportion of mutations being either strongly deleterious or nearly neutral, consistent with a *nearly neutral model* of molecular evolution (Ohta, 1973). Two expected results were also confirmed: synonymous substitutions had far smaller effects on fitness than did nonsynonymous ones (but see also Agashe et al., 2016; Cuevas, Domingo‐Calap, & Sanjuán, 2012; Zwart et al., 2018), and hydrophobic residues were more interchangeable with each other than were polar ones.

The interest in characterizing genotype–fitness maps for other systems was immediately recognized, and experiments have now been performed on genes encoding a diverse range of functions including ubiquitin, poly(A)‐binding protein, antibiotic resistance enzymes, a small nucleolar RNA, tRNAs, metabolic enzymes and regulatory regions (Bernet & Elena, 2015; Chan, Venev, Zeldovich, & Matthews, 2017; Dandage et al., 2018; Domingo, Diss, & Lehner, 2018; Firnberg, Labonte, Gray, & Ostermeier, 2014; Jacquier et al., 2013; Klesmith, Bacik, Michalczyk, & Whitehead, 2015; Li, Qian, Maclean, & Zhang, 2016; Li & Zhang, 2018; Melamed, Young, Gamble, Miller, & Fields, 2013; Melnikov, Rogov, Wang, Gnirke, & Mikkelsen, 2014; Puchta et al., 2016; Roscoe, Thayer, Zeldovich, Fushman, & Bolon, 2013; Wrenbeck, Azouz, & Whitehead, 2017). Due to the ease of massively parallel genotype–fitness mapping with this approach, many artificial systems have been devised in which fitness is used as a readout of some other phenotype of interest, such as gene expression (Shultzaberger et al., 2012; Shultzaberger, Malashock, Kirsch, & Eisen, 2010), protein binding affinity (Diss & Lehner, 2018) or protein stability (Kim, Miller, Young, & Fields, 2013). Caution should therefore be taken in comparing studies, as nonlinearities between different phenotypic levels likely have a major influence on the observed properties of the genotype–phenotype relationship, as will be discussed below.

This last decade has thus provided a rich pool of experimental data with which to explore statistically the genotype–phenotype relationship across different scales, and the following will summarize the emerging trends.

### 2.2 Distribution of mutational effects (DMEs) in single genes

The distribution of effects of individual new mutations is of profound importance to medical and evolutionary genetics (Eyre‐Walker & Keightley, 2007). Understanding it can also help guide strategies for directed evolution of useful biomolecules. The deep mutational scanning studies outlined above have revealed, perhaps unsurprisingly, that it differs between coding and noncoding regions, different types of genes and even different regions within genes.

#### 2.2.1 The DME in single proteins

**Figure 2:**
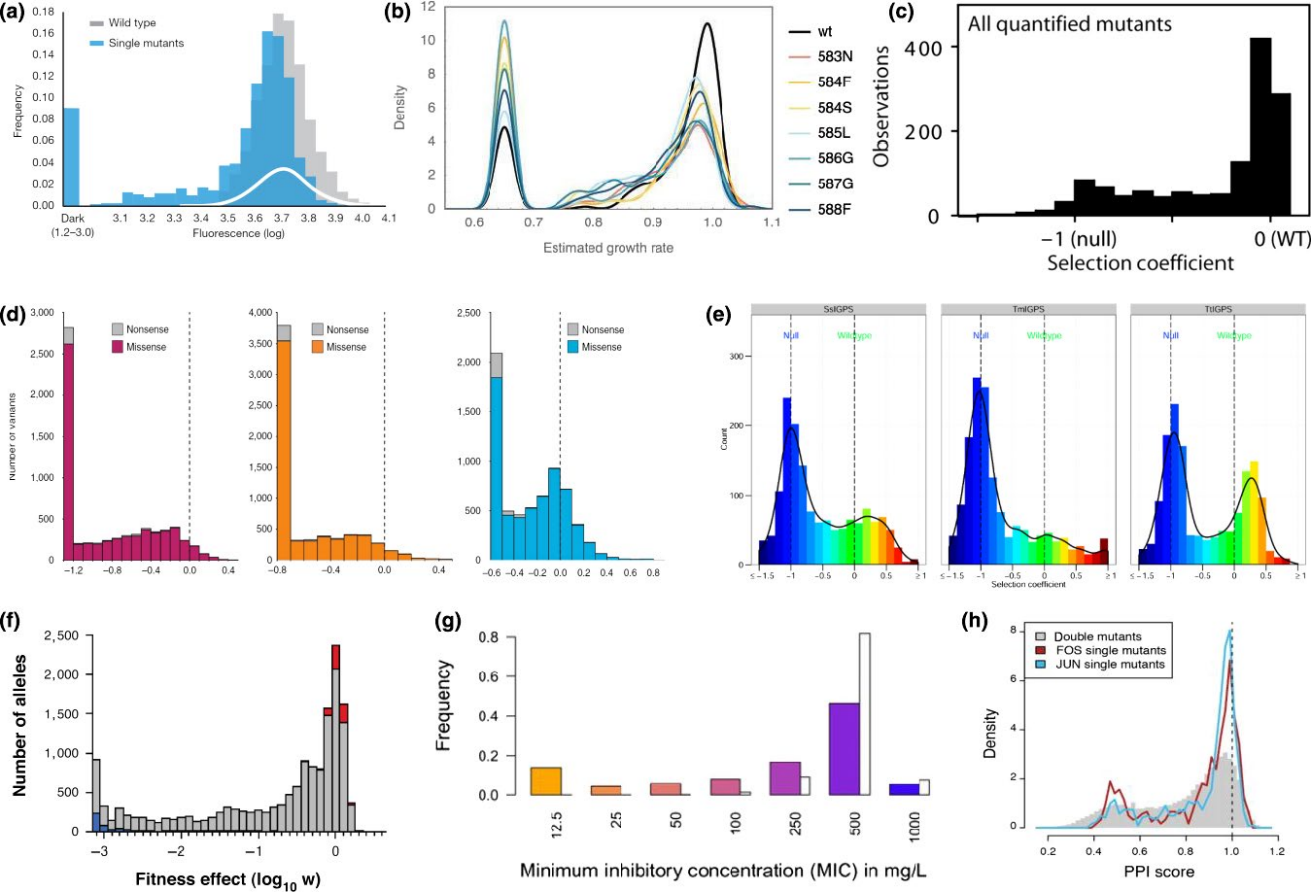
A sample of experimentally characterized distributions of mutational effects (DMEs) in various proteins, all showing at least 2 modes. (a) Distribution of fluorescence intensities resulting from single mutations across the length of a green fluorescent protein (blue). [Reprinted by permission from Springer Nature: *Nature*, Local fitness landscape of the green fluorescent protein (Sarkisyan et al., 2016), Copyright 2016]. (b) Distributions of yeast growth rate effects of all single point mutations in a 9‐amino‐acid region of 8 variants of a native chaperone protein (the wild‐type (black) and 7 single‐mutant variants). [Reprinted by permission of the Society for Molecular Biology and Evolution: *Molecular Biology and Evolution, 32*(1), 232, A systematic survey of an intragenic epistatic landscape (Bank et al., 2015)]. (c) Distribution of yeast fitness effects of all single point mutations across the length of native ubiquitin. [Reprinted from *Journal of Molecular Biology*, *425*(8), 1,366, Analyses of the effects of all ubiquitin point mutants on yeast growth rate (Roscoe et al., 2013). Copyright (2013), with permission from Elsevier]. (d) Distributions of bacterial fitness effects of all single point mutations across the length of a non‐native metabolic enzyme, on which the host strain has been made dependent for nitrogen supply, in the presence of 3 different amide substrates. [Reprinted from (Wrenbeck et al., 2017), licensed under CC BY 4.0]. (e) Distributions of yeast fitness effects of single mutations in the β‐barrel core region of 3 phylogenetically divergent orthologues of a metabolic enzyme, on which the host strain has been made dependent for tryptophan biosynthesis. [Reprinted from (Chan et al., 2017), licensed under CC BY 4.0]. (f) Distribution of “gene fitness” effects of all single codon substitutions across the length of a native bacterial antibiotic resistance gene, whose product's cellular activity is linked to fitness *via* a synthetic genetic circuit (grey: missense, blue: nonsense, red: synonymous). [Reprinted by permission of the Society for Molecular Biology and Evolution: *Molecular Biology and Evolution, 31*(6), 1583, A comprehensive, high‐resolution map of a gene's fitness landscape (Firnberg et al., 2014)]. (g) Distribution of minimum inhibitory concentrations of antibiotic observed for random single point mutations across the length of a native bacterial antibiotic resistance gene (same as f) (coloured bars; white bars: wild type). [Reprinted from *Proceedings of the National Academy of Sciences*, *110*(32), 13,068, Capturing the mutational landscape of the beta‐lactamase TEM‐1 (Jacquier et al., 2013)]. (h) Distributions of complementation assay protein–protein interaction scores resulting from single point mutations in the leucine zipper domains of 2 human transcription factor subunits (red and blue). [Reprinted from (Diss & Lehner, 2018), licensed under CC BY 4.0]

**Figure 3:**
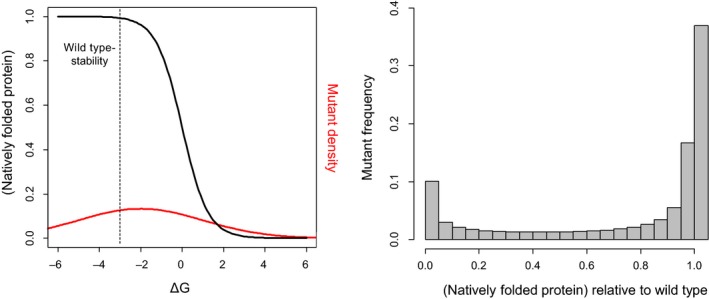
Illustration of the thermodynamic hypothesis for DMEs in proteins. Left panel—Black sigmoid curve shows the fraction of natively folded protein molecules as a function of the free energy of folding, *ΔG*, following: $P^{\mathrm{nat}}=\frac{1}{1+e^{\Delta G/k_{b}T}}$, where *k_b_* is the Boltzmann constant and *T* is temperature (*k_b_T* is set here to 0.62, as in (Wylie & Shakhnovich, 2011)). Dashed line marks a hypothetical wild‐type protein stability (−3 kcal/mole), located on the plateau of the sigmoid, for illustration. Red curve shows a hypothetical distribution of mutant *ΔG* values, resulting from a DME on *ΔG* that is Gaussian with a mean of +1, following (Wylie & Shakhnovich, 2011), but here with a larger standard deviation of 3. The stability sigmoid could be steepened by effects such as irreversible aggregation or degradation of misfolded species (Tokuriki & Tawfik, 2009). Right panel: The resulting DME on the *relative fraction of natively folded protein molecules*, which is bimodal under these parameter values

In proteins, whether the focal phenotype is biochemical functionality (Lagator, Sarikas, Acar, Bollback, & Guet, 2017; McLaughlin et al., 2012; Sarkisyan et al., 2016) or a highly integrated trait like fitness (Bank, Hietpas, Jensen, & Bolon, 2015; Bank, Hietpas, Wong, Bolon, & Jensen, 2014; Chan et al., 2017; Diss & Lehner, 2018; Firnberg et al., 2014; Hietpas et al., 2011; Jacquier et al., 2013; Jiang et al., 2016; Jiang, Mishra, Hietpas, Zeldovich, & Bolon, 2013; Klesmith et al., 2015; Mavor et al., 2016; Melamed et al., 2013; Melnikov et al., 2014; Roscoe et al., 2013; Wrenbeck et al., 2017), the DME appears to be universally multimodal, typically with near‐neutral and highly deleterious modes and a vanishing fraction of beneficial effects (Figure 2). An almost trivial exception to this is the DME of repressor proteins on expression (Lagator, Sarikas, et al., 2017), or more generally when a decrease in one phenotype leads to an increase in a downstream phenotype. The same multimodality is found for random mutation samples across whole viral genomes, which are dense with protein‐coding sequences (Carrasco, de la Iglesia, & Elena, 2007; Domingo‐Calap, Cuevas, & Sanjuán, 2009; Peris, Davis, Cuevas, Nebot, & Sanjuan, 2010; Sanjuan, Moya, & Elena, 2004b). A popular and conceptually simple mechanistic hypothesis for this is as follows: (a) a globally determined property of proteins is stability; (b) stability is potentially affected by amino‐acid changes at many positions, while positions contributing to a limiting step in direct protein function are likely to be rare; (c) protein folding is cooperative, resulting in a “thermodynamic cliff” in the stability–folding function; (d) protein mutants therefore tend to lie either at the plateau at the top of this cliff (where the majority of molecules are correctly folded), which is likely where the wild‐type resides, or at the bottom of it (majority of molecules unfolded); and (e) the concentration of natively folded molecules is likely a key determinant of protein activity, and the phenotype being measured depends either directly or indirectly on this activity (Bank et al., 2015; Firnberg et al., 2014; Jacquier et al., 2013; Olson, Wu, & Sun, 2014; Sarkisyan et al., 2016; Tokuriki & Tawfik, 2009; Wylie & Shakhnovich, 2011) (Figure 3).

**Figure 4:**
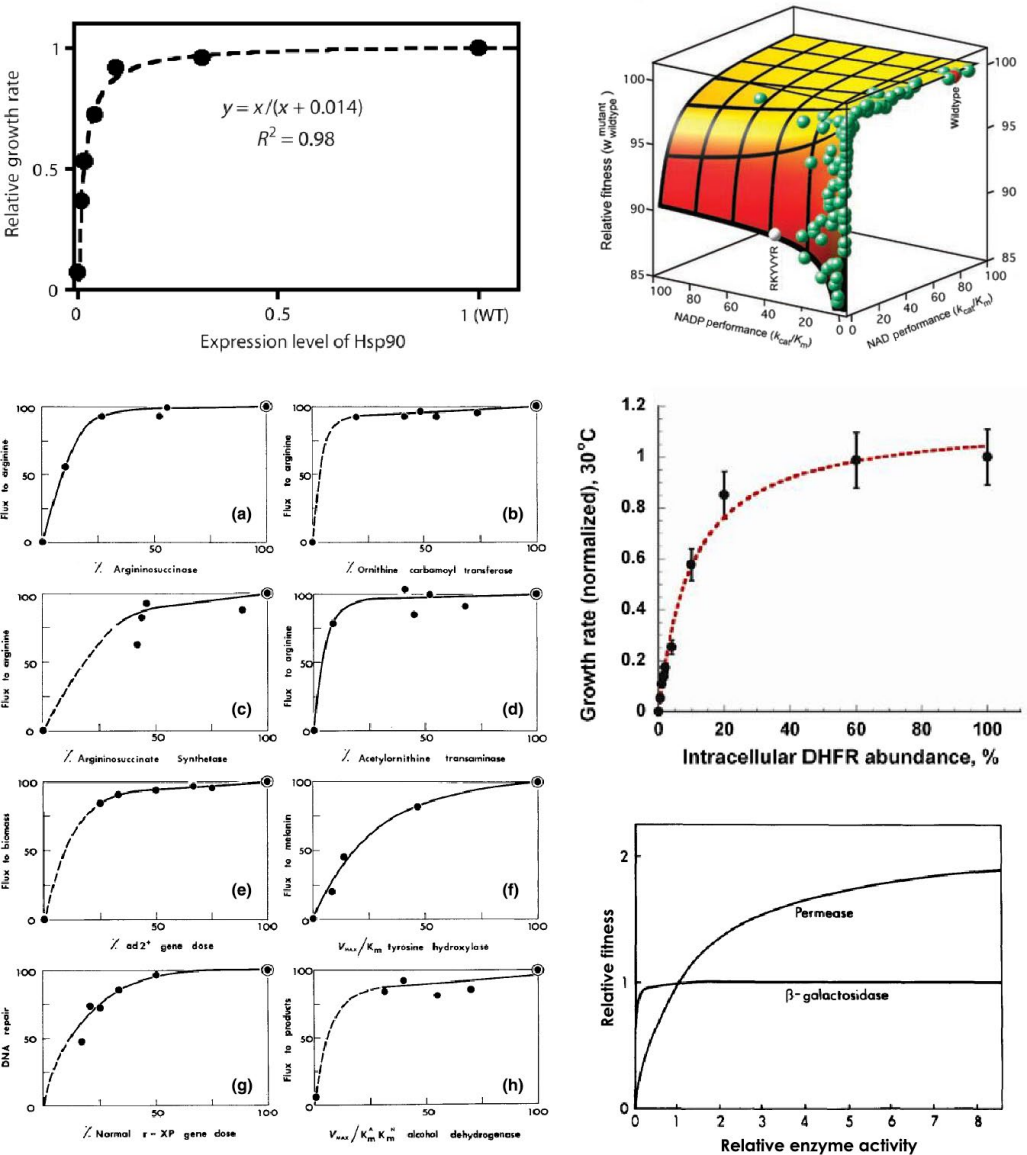
A sample of experimentally characterized elasticity functions, all of a saturating concave form. (a) Function: expression level—growth rate; protein: chaperone; organism: *Saccharomyces cerevisiae*. [Reprinted from (Jiang et al., 2013), licensed under CC BY 4.0]. (b) Function: enzymatic performance (*k*_cat_/*K*_m_)—fitness, under 2 different coenzymes**;** protein: oxidoreductase (amino‐acid biosynthesis); organism: *Escherichia coli*. [From *Science*, *310*(5,747), 501, The biochemical architecture of an ancient adaptive landscape (Lunzer et al.., 2005). Reprinted with permission from AAAS]. (c) Left‐right, top‐bottom. Functions: enzymatic activity—metabolic flux (first 4), gene dose—growth rate, enzymatic efficiency (*V*_max_/*K*_m_)—metabolic flux, gene dose—DNA repair rate, enzymatic efficiency—metabolic flux**;** proteins: lyase, transferase, ligase, aminotransferase (all from same amino‐acid biosynthesis pathway), carboxylase (nucleotide biosynthesis), oxidoreductase (melanin biosynthesis), unknown gene defective in a *xeroderma pigmentosum* patient (nucleotide repair), oxidoreductase (ethanol oxidation); organism: *Neurospora crassa* (first 4), *Saccharomyces cerevisiae*, *Mus musculus*, *Homo sapiens*, *Drosophila melanogaster*. [Republished with permission of Genetics Society of America, from *Genetics*, *97*(3–4), 642, The molecular basis of dominance (Kacser & Burns, 1981); permission conveyed through Copyright Clearance Center, Inc.]. (d) Function: expression level—growth rate**;** protein: oxidoreductase (cofactor biosynthesis); organism: *Escherichia coli*. [Reprinted from *Molecular Cell*, *49*(1), 137, Protein quality control acts on folding intermediates to shape the effects of mutations on organismal fitness (Bershtein et al., 2013). Copyright (2013), with permission from Elsevier]. (e) Function: enzymatic activity—fitness**;** proteins: sugar:proton symporter and hydrolase (sugar catabolism); organism: *Escherichia coli*. [Republished with permission of Genetics Society of America, from *Genetics*, *115*(1), 29, Metabolic flux and fitness (Dykhuizen et al., 1987); permission conveyed through Copyright Clearance Center, Inc.]

Whatever the mechanism(s) responsible for changes in protein activity, however, the precise form of the distribution of mutational effects must also depend on the quantitative relationship between activity and the measured phenotype, and on the activity of the wild type. For example, three orthologous wild‐type indole‐3‐glycerol phosphate synthases were found to have their neutral mode shifted towards beneficial effects, showing they were sub‐optimal for fitness under the chosen experimental conditions (Chan et al., 2017) (Figure 2e). A striking demonstration of the impact of a nonlinear activity–fitness relationship was provided by the Hsp90 chaperone protein (Jiang et al., 2013), found to have a concave, saturating activity–fitness function. Interestingly, similarly shaped functions are found to generally describe activity–*flux* or activity–fitness relationships across enzymes and organisms, in line with expectations from metabolic control analysis (Bershtein, Mu, Serohijos, Zhou, & Shakhnovich, 2013; Dykhuizen, Dean, & Hartl, 1987; Jiang et al., 2013; Kacser & Burns, 1981; Lunzer, Miller, Felsheim, & Dean, 2005) (Figure 4). As is often found, wild‐type Hsp90 activity lays safely on this plateau, far from the fitness shoulder, at endogenous expression levels. This meant that even mutations with an intermediate effect on *activity* could be nearly neutral to fitness. By then measuring the distribution of fitness effects at increasingly reduced expression levels, these latent activity effects were increasingly revealed. Indeed, the large number of mutations found to have intermediate, rather than nearly neutral, effects on activity suggested that the dominant mechanism for activity changes was *via* specific molecular function (substrate binding), rather than global stability.

**Figure 5:**
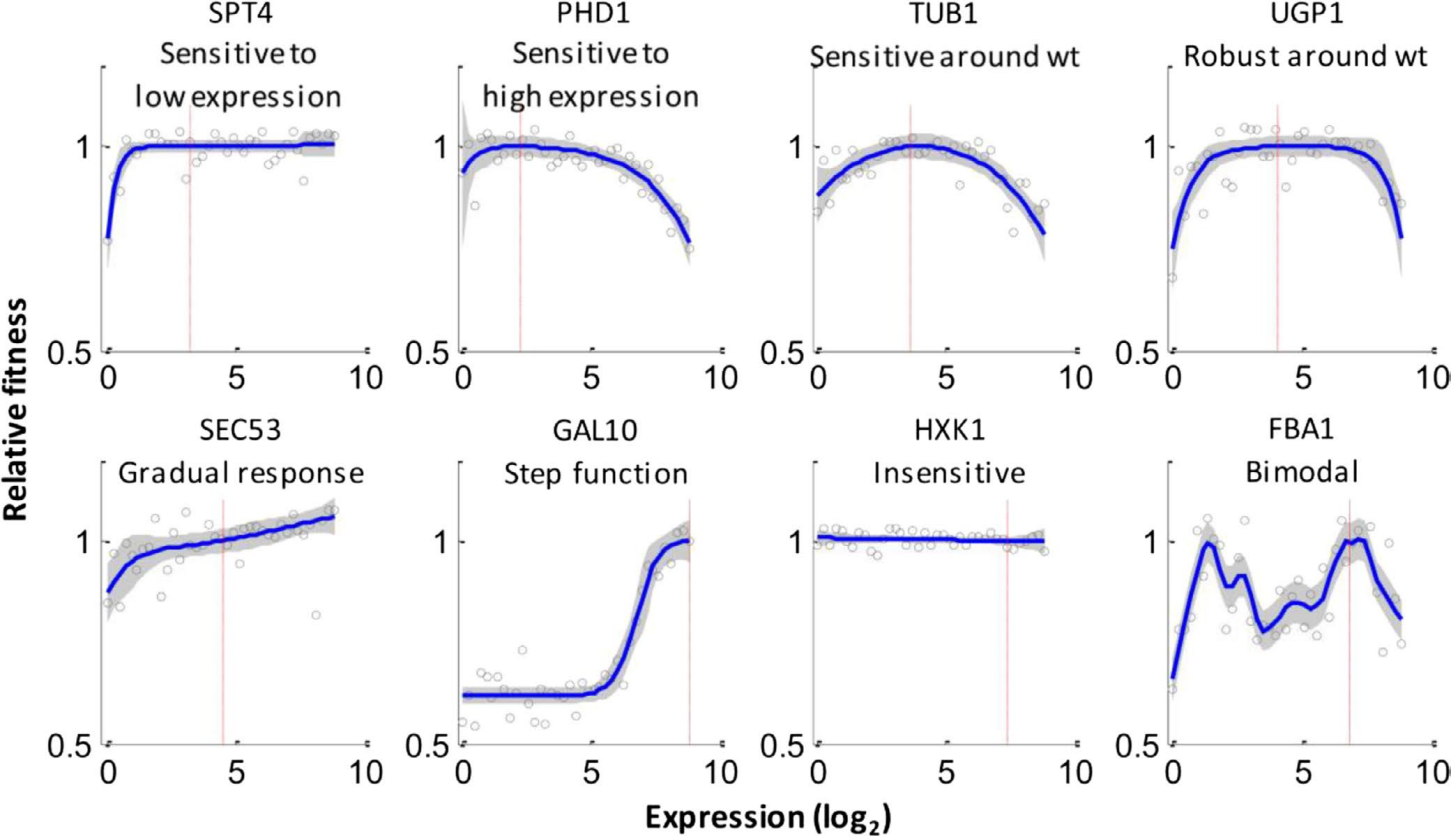
Expression–fitness functions for a diverse set of protein‐coding yeast genes. Red lines mark wild‐type expression levels. [Reprinted from *Cell*, *166*(5), 1,286, Massively parallel interrogation of the effects of gene expression levels on fitness (Keren et al., 2016). Copyright (2016), with permission from Elsevier**]**

The DME in proteins, as will be found in other sequences, is thus shaped by forces at several scales, from the intramolecular level to the intermolecular interface level to the cell‐system level and beyond. A source of bias to be wary of is therefore the choice of experimental system: it is often desirable to focus on systems suspected to show linear, or at least monotonic, relationships across these levels (*e.g*. the activity–fitness function), but more complex relationships may well be common (Baeza‐Centurion, Miñana, Schmiedel, Valcárcel, & Lehner, 2019; Hsin‐Hung Chou, Delaney, Draghi, & Marx, 2014; Dekel & Alon, 2005; Drummond & Wilke, 2008; Perfeito, Ghozzi, Berg, Schnetz, & Lässig, 2011; Rokyta et al., 2011; Serohijos, Rimas, & Shakhnovich, 2012; Serohijos & Shakhnovich, 2014; Shultzaberger et al., 2010; Towbin et al., 2017). A clear demonstration of this was provided recently for the case of *expression–*fitness relationships—these were characterized in parallel for 81 diverse genes in the yeast, *S. cerevisiae*, by inserting in front of each gene a library of synthetic promoters of known strength (Keren et al., 2016). In addition to the commonly found concave function, a variety of other forms were uncovered, including step‐like ones, flat ones and single‐ or multipeaked ones (Figure 5). Importantly, genes from the same pathway or complex tended to display similar expression–fitness curves, suggesting that these are shaped primarily by the cell‐level function of a gene, rather than its specific biochemistry. The consequences of these different elasticity functions for the DME across different genes should therefore be a fruitful avenue for future research.

#### 2.2.2 The DME in single functional noncoding RNAs

The DME for the few functional noncoding RNA sequences (*cis*‐regulatory region, tRNA, snoRNA, twister ribozyme) so far examined appears to follow similar rules to proteins: deleterious and nearly neutral modes and a miniscule proportion of beneficial mutations again probably shaped in part by both a thermodynamic stability threshold and common saturating concave activity–fitness functions (Bendixsen, Østman, & Hayden, 2017; Bernet & Elena, 2015; Kobori & Yokobayashi, 2016; Li et al., 2016; Puchta et al., 2016). This is perhaps not surprising because (a) as for proteins, noncoding RNA function depends strongly on structure, and (b) there is no obvious reason why the dependence of cell fitness on RNA‐regulated activities or direct RNA activities (especially those chosen for experimental investigation) would be fundamentally different to its dependence on protein activities.

#### 2.2.3 The DME in single cis‐regulatory DNA regions

In the case of *cis*‐regulatory DNA sequences, measured DMEs are generally unimodal (Badis et al., 2009; Kinney et al., 2010; Lagator, Igler, Moreno, Guet, & Bollback, 2016; Lagator, Sarikas, et al., 2017; Warren et al., 2006), although they can be multimodal in more complex regulatory contexts (Lagator, Paixão, Barton, Bollback, & Guet, 2017; Lagator, Sarikas, et al., 2017) or when a fraction of the sites function through specific base‐pairing with other nucleic acids (and so are highly sensitive to mutation; Boyle et al., 2017). The majority of mutations decrease DNA binding to regulators (again presumably because wild‐type sequences are optimized for relatively strong binding), but of course the impact of this on downstream phenotypes depends if the regulation is activational, repressive or some complex mixture of both (Lagator, Paixão, et al., 2017). The biophysical reason for a more uniform DME on direct biochemical phenotypes for *cis*‐regulatory DNA sequences than for proteins and noncoding RNAs is not clear: the thermodynamics of DNA–protein binding can result in similar energy–phenotype functions to those of macromolecular folding described earlier (Lagator, Paixão, et al., 2017; Mustonen, Kinney, Callan, & Lassig, 2008; Vilar, 2010). It may be that in reality they are less steep (e.g. because deleterious effects from misfolded species no longer contribute) or that *cis*‐regulatory mutations tend to have smaller effects on binding energy than do protein and noncoding RNA mutations on folding energy, and so sample a smaller region of the energy space (see Figure 3).

### 2.3 Epistasis within single genes (intragenic epistasis)

**Figure 6:**
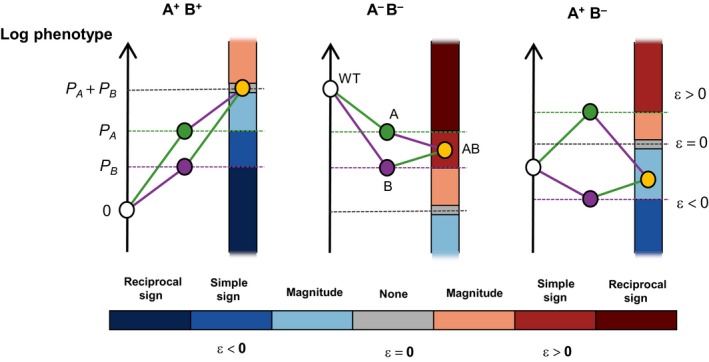
Categories of epistasis possible for different types of mutation pairs. “A” and “B” are mutations, and superscript “+” and “−” denote that these individual mutations increase or decrease the value of the measured phenotype, *P*. In all cases, the white point is wild type and the orange point is the AB double mutant. Grey dashed line marks the sum of *P*_A_ and *P*_B_, that is the *expected* value for the double mutant. Epistasis measures the deviation from this expectation, which may be either negative or positive, and can be categorized as either magnitude (the direction of mutational effects do not depend on the other mutation) or sign type. Sign epistasis can be further categorized as simple (effect of one mutation changes sign in the presence of the other) or reciprocal (effects of both mutations change sign in the presence of the other). The three examples shown are (left‐right): no epistasis between a pair of positive‐effect mutations, positive simple sign epistasis between a pair of negative‐effect mutations, and negative magnitude epistasis between a positive‐effect and negative‐effect mutation

Epistasis is defined here as the deviation of an observed phenotype value from that expected if the constituent individual mutations combined additively on the log scale (*i.e*. multiplicatively on the linear scale; Wolf, Brodie III, & Wade, 2000) (Figure 6). Epistasis is critically important in medical and evolutionary genetics and bioengineering: among other things, it confounds prediction of mutational effects, constrains adaptive paths, determines the benefit of sex and hinders efforts to increase yields and activities of industrially useful substances (Badano & Katsanis, 2002; Breen, Kemena, Vlasov, Notredame, & Kondrashov, 2012; Dipple & McCabe, 2000; Hansen, 2013; Kimura & Maruyama, 1966; Kondrashov, 1988; Kondrashov & Kondrashov, 2001; Manolio et al., 2009; Niederberger, Prasad, Miozzari, & Kacser, 1992; Phillips, 2008; Scriver & Waters, 1999; Weinreich, 2006). Intragenic epistasis is logically shaped by the same forces as those shaping the DME, such as thermodynamics and phenotype–phenotype functions (Lehner, 2011; de Visser, Cooper, & Elena, 2011), and its basic statistical properties will now be summarized for the systems in which large‐scale measurements have been made.

#### 2.3.1 Intragenic epistasis within single proteins

**Figure 7:**
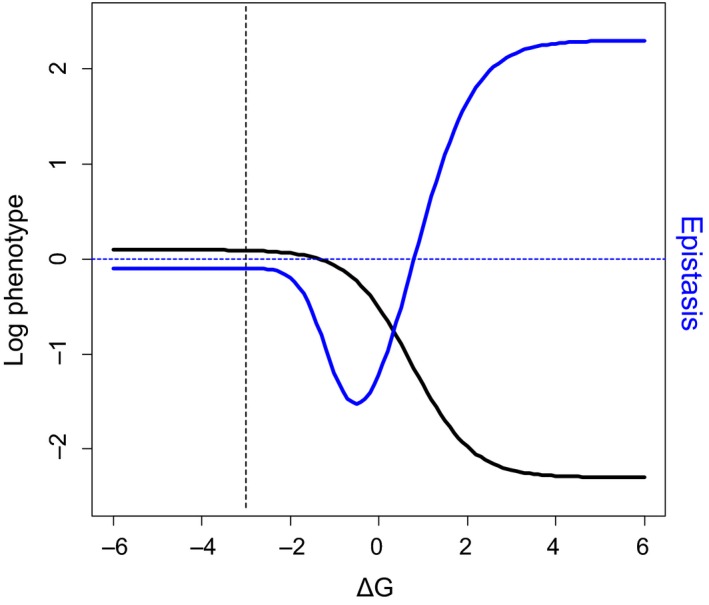
Trends of epistasis predicted by thermodynamic model of mutation effects. Black sigmoid curve shows the logarithm of a phenotype that increases proportionally with the fraction of natively folded protein molecules as a function of the free energy of folding, *ΔG*. A small background value (phenotype of 0.1 in the absence of any correctly folded molecules) has been applied to capture the situation for nonessential genes and/or the effect of measurement background/limits. If the phenotype truly approaches 0 in the limit of very high *ΔG*, the stability curve is no longer sigmoidal on this log scale, but has a concave shape, causing epistasis (see below) to become increasingly negative, in a linear fashion, as *ΔG* increases. In reality, however, biology or experimental limitations often result either in a background phenotype value in the absence of correctly folded protein, resulting in a log‐sigmoid as shown here, or in a threshold being applied below which all mutants are considered null and therefore not considered for epistasis analysis. The formula and parameter values are as for Figure 3, with the addition of the 0.1 phenotype background. Dashed vertical line marks a hypothetical wild‐type protein stability (−3 kcal/mole), located on the stability plateau. Blue curve shows the epistasis that would occur between pairs of mutations of identical *ΔG* effects, each of which individually displaces *ΔG* from the wild‐type value to the value indicated by the *x*‐axis. Dashed horizontal line marks the boundary between positive and negative epistasis (*i.e*. zero epistasis). A transition from negative to positive epistasis occurs as mutations become more strongly destabilizing, due to the sigmoidality of the stability curve. The shape of the epistasis curve could explain why both negative and positive epistasis are observed between mutations within proteins, as well as the existence of certain correlations between mutation effect size and epistasis (see below)

Within proteins, epistasis appears prevalent and predominantly negative, with a unimodal distribution. As the majority of mutations tend to reduce the value of the measured phenotype, this epistasis mainly represents *synergistic* interactions between deleterious mutations: the combined impact of multiple mutations is often *worse* than “the sum of the parts” (Bank et al., 2015; Bank, Matuszewski, Hietpas, & Jensen, 2016; Melamed et al., 2013; Olson et al., 2014; Sarkisyan et al., 2016) (but Araya et al., 2012; Diss & Lehner, 2018 are exceptions). Under the stability threshold model outlined above, such synergistic negative epistasis would indeed be expected to prevail between mutations having mildly destabilizing effects, because, beginning from the stability plateau, the downward slope initially becomes steeper as stability is decreased. After a threshold is crossed, however, the slope levels off again, which can result in positive epistasis being detected between highly destabilizing mutations if the protein is nonessential or if an experimental measurement limit is approached (Bank et al., 2015; Bershtein, Segal, Bekerman, Tokuriki, & Tawfik, 2006; Diss & Lehner, 2018; Jacquier et al., 2013; Sarkisyan et al., 2016; Wylie & Shakhnovich, 2011; Figure 7). Differences in the destabilizing effects of mutations, wild‐type stability and measurement precision/range could therefore explain discrepancies between studies as to the pervasiveness of epistasis as well as the relative fractions of positive and negative interactions. Negative epistasis between mutations of mildly deleterious biochemical effect could also result from concave saturating elasticity functions (described above) (Bank et al., 2015; Szathmary, 1993), for the same reason that it results from the stability function, and analogously to the classical molecular hypothesis of genetic dominance and recessivity (Kacser & Burns, 1981). Of course, as for the DME, many specific cases will deviate from these general expectations and vary across proteins. For example, specific structural interactions could generate sign epistasis when mutations do not act additively on the level of *folding energy* (*ΔG*) (Otwinowski, McCandlish, & Plotkin, 2018; Starr & Thornton, 2016). Further, any single mutation could potentially effect multiple molecular phenotypes, including protein stability, affinity, activity (existing or new) per folded species, folding and assembly kinetics, aggregation propensity, degradation rate and post‐translational modification, as well as RNA splicing (Baeza‐Centurion et al., 2019; DePristo, Weinreich, & Hartl, 2005; Echave & Wilke, 2017; Otwinowski, 2018; Shah, McCandlish, & Plotkin, 2015; Sikosek & Chan, 2014), so simplistic models should be treated with caution. Indeed, a recent study demonstrated how mRNA secondary structure phenotypes affecting translation efficiency can have fitness consequences through a general, cell‐level mechanism of ribosome sequestration (Cambray, Guimaraes, & Arkin, 2018).

In addition to prevailing negative epistasis, a trend of “increasing losses” is sometimes detected in proteins (at least for mildly deleterious mutations), further supporting the idea that there exists some source of concavity in the phenotypic landscape (Bank et al., 2015; Diss & Lehner, 2018; Sarkisyan et al., 2016). This trend manifests as a positive correlation between the magnitude of individual mutation effects and the magnitude of epistasis they experience when combined. The flip side of “increasing losses” between the predominantly deleterious mutations of the DME is “diminishing returns” between the rare mutations from the beneficial tail of the DME (MacLean, Perron, & Gardner, 2010; Nagel, Joyce, Wichman, & Miller, 2012; Schenk, Szendro, Salverda, Krug, & de Visser, 2013): synergistic negative epistasis increases with the *increasing* downward slope moving away from the plateau, and antagonistic negative epistasis increases with the *decreasing* upward slope. The evolutionary outcome of these trends should be a more rapid purging of deleterious mutation combinations (negative selection), but a slower rate of adaptation resulting from beneficial combinations (positive selection), than would be expected in the absence of epistasis.

#### 2.3.2 Intragenic epistasis within functional noncoding RNAs

Epistasis between mutations residing in the same noncoding RNA molecule, as for the DME and again probably for the same reasons, appears similar to the case of proteins: it is common and usually biased towards negativity (Bendixsen et al., 2017; Domingo et al., 2018; Kobori & Yokobayashi, 2016; Li et al., 2016; Chuan Li & Zhang, 2018; Puchta et al., 2016). These experimental results are in contrast to the predominance of positive epistasis predicted by earlier computational RNA folding models (Wilke & Christoph, 2001; Wilke, Lenski, & Adami, 2003), but it is not clear whether this is due to the binary nature of these models or the fact that they do not use naturally evolved sequences as their starting point (Bendixsen et al., 2017). When higher‐order epistasis was examined experimentally, positive and negative epistasis were found to be equally prevalent, with many cases of sign epistasis, suggesting a rugged multidimensional fitness landscape (Domingo et al., 2018; *cf*. Bank et al., 2016) for a protein region).

#### 2.3.3 Epistasis within single cis‐regulatory DNA regions

Epistasis within *cis*‐regulatory DNA has also been studied experimentally in a limited number of contexts. Within a mammalian *Rhodopsin* promoter region bound by at least two transcription factors, epistasis for expression was found to be biased towards negativity (Kwasnieski, Mogno, Myers, Corbo, & Cohen, 2012). More strikingly, in a recent study of the direct effect of target‐site mutations on dCas9‐DNA binding, negative epistasis for initial association rate between the universally deleterious single mutations was found to be ubiquitous—binding was essentially always worse than would be predicted from the simple addition of individual mutation effects (Boyle et al., 2017). Cas9‐DNA binding may be rather unrepresentative, however, as its function is clearly not regulatory (it is immune), and it is mediated by nucleic acid base‐pairing. Another team recently examined the expression epistasis between mutations within both the same and different operators in two different promoters: the *E. coli araBAD* promoter (Lagator et al., 2016) and the lambda bacteriophage P_R_ promoter (Lagator, Paixão, et al., 2017). Although these two promoters possess substantially different architectures, both exhibited a predominance of negative epistasis, whether mutations resided in the same or different operators and whether active repressor proteins were present in high concentration or not. For the simple case where expression is determined by DNA binding to a single regulator, this is to be expected from thermodynamic considerations: the free binding energy–expression curve resulting from their generic model is, once again, of a concave, saturating form (Lagator, Paixão, et al., 2017). Notably, though, in the lambda bacteriophage P_R_ promoter, the fraction of positive interactions increased when active repressor concentration was increased, as did the frequency of the most extreme form of interaction, reciprocal sign epistasis (from 8% to 66%). This is explained by the fact that repressor binding sites (operators) and RNA polymerase binding sites overlap in this promoter, and so, promoter mutations will tend to effect binding to both of these—one of which decreases transcription, the other of which increases transcription (Lagator, Paixão, et al., 2017).

*Cis*‐regulatory DNA sequences have thus proved to be an excellent model system for studying how epistasis can arise through the inherent *molecular* pleiotropy of mutations: they directly affect multiple molecular phenotypes (here, binding energy with different regulatory proteins), often differentially, and the *measured* phenotype (here, expression) is then some function of these multiple input phenotypes. In reality, as for proteins and noncoding RNAs, many more molecular phenotypes than those typically considered are potentially impacted by an individual mutation, such as DNA structure (Rajkumar, Dénervaud, & Maerkl, 2013), binding site accessibility (Levo & Segal, 2014), regulator protein cooperativity (Todeschini, Georges, & Veitia, 2014) and long‐range DNA looping (Levine, Cattoglio, & Tjian, 2014), likely accounting for the significant fraction of observed interactions that are not explained by simple binding energy models (Lagator, Paixão, et al., 2017). Current empirical studies thus present a minimal mechanistic picture of the potential sources of epistatic interactions, and the goal now should be to find additional molecular phenotypes that can account for some of the unexplained epistasis.

Finally, *cis*‐regulatory sequences appear to play a major role in evolutionary processes (Wittkopp & Kalay, 2012; Wray, 2007), yet none of the studies above have assessed epistasis at the level of fitness. As for proteins and RNA, in addition to the mechanisms just discussed, this is bound to be also shaped by the expression–fitness elasticity functions of the regulated genes. Fortunately, these are far more accessible to high‐throughput genome‐wide characterization than are pure activity–fitness functions (Keren et al., 2016).

## 3 TWO GENES

Adding just one more gene, let alone several, to the kinds of high‐throughput studies that have provided such rich insights for single genes generally requires more than simply twice the effort (“experimental effort epistasis”). For many systems, the problem is that the length of DNA fragments required to carry two different genes exceeds those amenable to the highest‐throughput sequencing technologies (max. ~1kb with Illumina). Presumably largely for this reason, very few deep mutational scanning studies have assayed more than one gene or even regulatory sequence simultaneously, but unique DNA barcodes that allow a long sequence to be broken up for sequencing and then reassembled in silico provide one workaround (Fowler, Stephany, & Fields, 2014; Sarkisyan et al., 2016).

### 3.1 Epistasis between two genes (intergenic epistasis)

#### 3.1.1 Intergenic epistasis between two physically interacting partners

A recent exception was the measurement of intermolecular epistasis between a library of point mutants of the leucine zipper domains of two proto‐oncogenes, FOS and JUN (Diss & Lehner, 2018). The products of these two genes physically associate through these domains to form a transcription factor complex, AP‐1. In this case, the technical challenge of long sequencing fragments was overcome by isolating the two small leucine zipper domains, which are presumed to function in a modular manner, and cloning them adjacent to each other in the absence of the rest of the native proteins. An artificial complementation assay, in which intermolecular FOS‐JUN binding drives the assembly of a drug‐resistance enzyme, was used to link binding strength to cell growth in the presence of the drug. The relationship between abundance of the complementation complex and growth rate is well‐characterized and expected to be approximately linear. Intermolecular epistasis was found to be common and just slightly biased towards negativity. Importantly though, a characteristic relationship between the effect of single mutations and the interaction they experienced when combined suggested that the epistasis could be partly explained by a sigmoidal thermodynamic fitness landscape describing intermolecular binding (Figure 7). Indeed, applying this model removed the correlation between individual effects and epistasis, and increased the percentage of explained variance in double‐mutant phenotype scores from ~86% (under a simple multiplicative model) to ~89%. It may, however, be a particularity of the system, as the folding of leucine zippers like FOS and JUN is known to be coupled to their binding (Patel, Abate, & Curran, 1990; Thompson, Vinson, & Freire, 1993); *cf.* the 3‐state biophysical model of (Otwinowski, 2018)—studies on other pairs of binding partners are therefore necessary.

An impressive effort was also recently made to characterize the epistasis between a large number of mutants of a particular repressor protein and its *cis*‐regulatory DNA target at low‐throughput, using a fluorescent reporter to measure expression (Lagator, Sarikas, et al., 2017). Epistasis was detected for about half of the 150 pairwise interactions tested, the majority of which were positive. As before, much, but not all, of this epistasis could be rationalized in terms of the promoter architecture, which contained overlapping binding sites for the mutated repressor protein and RNA polymerase, raising hope that a set of rules may exist that adequately predicts epistasis both within promoters, and between promoters and regulators, when promoter architecture is known.

#### 3.1.2 Intergenic epistasis between two functionally interacting partners

**Figure 8:**
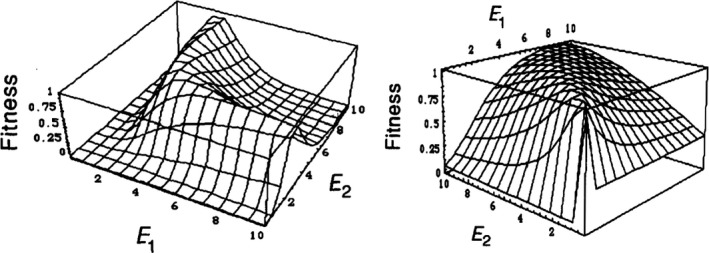
Two‐enzyme activity–fitness functions predicted from metabolic control analysis. E_1_ and E_2_ are the activities of two enzymes acting at adjacent steps of a linear metabolic pathway. In both plots, fitness is assumed to depend solely on the steady‐state concentration of a pathway intermediate, in a Gaussian manner (*i.e*. stabilizing selection is assumed to operate on the intermediate). The only difference is that, in one case, the intermediate lies downstream of the two enzymes (left), and in the other, it lies between them (right). The two landscapes have strikingly different forms, resulting in different expectations of interenzyme epistasis. Further, in both cases, trends of interenzyme epistasis will depend on the position of the wild type and the distribution of mutation effects on enzyme activities. [Republished with permission of Genetics Society of America, from *Genetics*, *133*(1), 129–130, Do deleterious mutations act synergistically? Metabolic Control Theory provides a partial answer (Szathmary, 1993); permission conveyed through Copyright Clearance Center, Inc.**]**

Although epistasis between protein–protein and protein–DNA binding partners is of great importance, the majority of genes in a given genome are expected to interact *indirectly*, through metabolic, regulatory and signalling networks. Metabolic networks are the most tractable of these, being based on simple mass flow. Metabolic control analysis (MCA) provides a rigorous framework to explore how pathway phenotypes such as flux and metabolite concentrations depend on the activity of several enzymes simultaneously, and thus enables predictions of interenzyme epistasis (Szathmary, 1993). Several small‐scale studies provide support for the validity of MCA in general (Hsin‐Hung Chou et al., 2014; Dykhuizen et al., 1987), but its rich predictions regarding the epistasis between genes connected through metabolic pathways remain untested. Importantly, the nature of fitness epistasis is predicted to vary considerably depending on which pathway phenotypes are under selection (flux, steady‐state metabolite levels), the pathway position of any selected metabolites relative to the two enzymes considered and the type of selection operating (directional, stabilizing) (Szathmary, 1993) (Figure 8). This points to the necessity of uncovering which phenotypes are typically under selection if we are to use such systems models to predict sequence–fitness relationships. Epistasis between two or more genes in signal‐flow networks has also never been tested on a systematic scale, but a recent small‐scale study on a synthetic gene regulatory cascade found a surprisingly high frequency of sign epistasis simply at the level of expression (briefly, explained by the fact that changes in the activity of one regulator shift the optimal activity of other regulators) (Nghe, Kogenaru, & Tans, 2018).

## 4 THE GENOME

### 4.1 Experimental approaches for genome‐wide genotype–phenotype mapping

**Figure 9:**
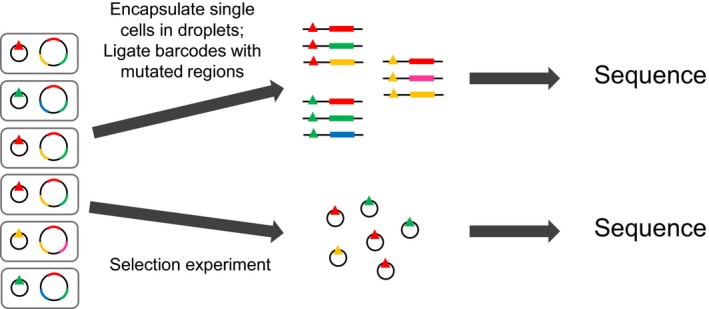
Barcoded‐Tracking of Combinatorial Engineered Libraries (bTRACE), a general high‐throughput method for analysing the effect of known genome‐wide mutation combinations. A multiplex genome‐engineering method is used to construct a cell library in which each clone can contain multiple mutations throughout the genome, and this library is itself transformed with a library of plasmids carrying highly diverse DNA barcodes (triangles), such that each cell now contains a unique barcode. Single cells are then encapsulated in emulsion droplets, where they are lysed, and a targeted binary PCR assembly reaction is performed to ligate barcodes adjacent to chosen genomic regions. The emulsion is broken, and deep‐sequencing of the assembled product pool allows reconstruction of the complete phased genotype associated with each barcode. In parallel, the library can be phenotyped by one of the deep‐sequencing techniques discussed previously, with only the small DNA barcodes now requiring sequencing, allowing genome‐wide mutation combinations to be linked to an amenable trait at high throughput. Figure based on Figure 1 from Zeitoun et al. (2017)

Scaling up deep mutational scanning experiments to the scale of the genome is at present out of reach: bottlenecks include the precise, genome‐wide introduction of individual mutations (mutagenesis efficiency and accuracy), sequencing costs and associating mutations at distant loci. The first is improving with advances in genome engineering, particularly from CRISPR‐Cas9‐based methods (Barbieri, Muir, Akhuetie‐Oni, Yellman, & Isaacs, 2017; Haimovich, Muir, & Isaacs, 2015; Roy et al., 2018). The second is continuing to improve, following a long‐term trend of decreasing costs (but see (Muir et al., 2016) for the alternative challenge of managing increasing amounts of data); and the third is becoming more feasible with emulsion‐based generalized DNA assembly technologies that encapsulate single cells and enable distal DNA sites to be linked by sequencing (either by directly ligating mutated sites adjacent to each other (Haliburton, Shao, Deutschbauer, Arkin, & Abate, 2017; Zeitoun et al., 2015) or, more scalably, by ligating them to a cell‐specific DNA barcode (Zeitoun, Pines, Grau, & Gill, 2017)) (Figure 9).

In the meantime, a plethora of “functional genomics” systematic genome‐wide studies have been performed, especially in yeast, that measure the effects (typically growth) of different types of large perturbations in single or multiple (maximum of 3) genes (deletion, overexpression, knockdown, transposon insertion; typically one or two perturbations per gene) (Baba et al., 2006; Babu et al., 2014; Boutros et al., 2004; Breslow et al., 2008; Collins et al., 2007; Costanzo et al., 2010, 2016; Davierwala et al., 2005; Decourty et al., 2008; Douglas et al., 2012; Fuchs et al., 2010; Gagarinova et al., 2016; Giaever et al., 2002; Jaffe, Sherlock, & Levy, 2017; Kamath et al., 2003; Kuzmin et al., 2018; Nichols et al., 2011; Onge et al., 2007; van Opijnen, Bodi, & Camilli, 2009; Roguev et al., 2008; Sameith et al., 2015; Schuldiner et al., 2005; Szappanos et al., 2011; Tischler, Lehner, Chen, & Fraser, 2006; Tong, 2004; Tsherniak et al., 2017; Ursell et al., 2017). These provide rich functional data sets, but there is little evidence that any genotype–phenotype inferences would generalize to the less extreme perturbations (*e.g*. point mutations) often found in nature (Rojas Echenique, Kryazhimskiy, Nguyen Ba, & Desai, 2019). Further, the majority are biased towards altering functions of known genes. An approach to study genome‐wide genotype–fitness relationships at the other extreme is mutation accumulation experiments and the analysis of natural DNA sequence data. These benefit from sampling *naturally occurring* mutations, which may be very different to those introduced experimentally, and potentially capturing mutations of effects too weak to be quantified directly, but they rely purely on inferences (Eyre‐Walker & Keightley, 2007). In between these two approaches are experiments that directly measure the effects of randomly induced or collected mutations (Bonhoeffer, 2004; Carrasco et al., 2007; Domingo‐Calap et al., 2009; Peris et al., 2010; Sanjuan et al., 2004b; Szafraniec, Wloch, Sliwa, Borts, & Korona, 2003; Wloch, Szafraniec, Borts, & Korona, 2001), or of mutations that are detected during experimental evolution (Caudle, Miller, & Rokyta, 2014; Chou, Chiu, Delaney, Segre, & Marx, 2011; Chou et al., 2014; Flynn, Cooper, Moore, & Cooper, 2013; Khan, Dinh, Schneider, Lenski, & Cooper, 2011; Kryazhimskiy, Rice, Jerison, & Desai, 2014; Kvitek & Sherlock, 2011; Rokyta et al., 2011; Venkataram et al., 2016), all of which provide data sets orders of magnitude smaller than do systematic perturbation studies.

### 4.2 The genome‐wide DME

An early finding of the genome‐wide perturbation studies was that, in most organisms and in permissive conditions, the majority of genes are inessential (Baba et al., 2006; Gerdes et al., 2003; Giaever et al., 2002; D.‐U. Kim et al., 2010; Sassetti, Boyd, & Rubin, 2003; Viswanatha, Li, Hu, & Perrimon, 2018; Yamamoto et al., 2009; Zhang & Lin, 2009). A notable exception is the “minimal bacterium”, *Mycoplasma genitalium* (Glass et al., 2006; Hutchison et al., 1999), which was in fact the first organism in which gene essentiality was examined directly (Hutchison et al., 1999), demonstrating a frequent irony in experimental biology: the first choice of experimental system is usually based on convenience, which in some cases results in it being utterly unrepresentative. “Chemical genomics” approaches, which phenotype genome‐wide perturbation libraries in many different defined environments, perhaps unsurprisingly, reduce the fraction of inessential genes by revealing *conditionally essential* genes that are necessary for growth in at least one of the environments tested (Nichols et al., 2011). The interpretation of these classifications (essential, conditionally essential, inessential) is not clear, however, as they are clearly fully dependent on the environments tested (see *Environment*).

Interestingly, it seems that the DFE of single‐gene deletions/disruptions (Baryshnikova et al., 2010; van Opijnen, Lazinski, & Camilli, 2014; Wang et al., 2018) might be qualitatively similar to the DFE of randomly sampled/induced genome‐wide mutations (Eyre‐Walker & Keightley, 2007), itself similar to the DFE of single point mutations in proteins and noncoding RNAs. These all comprise a nearly neutral mode with a heavy negative tail, a very deleterious/lethal mode (sometimes ignored) and a small proportion of beneficial effects (see (Bataillon & Bailey, 2014; Eyre‐Walker & Keightley, 2007) for more fine‐scale properties). Uncovering the reasons for this universality should prove to be a fascinating endeavour.

In the meantime, top‐down heuristic phenotype–fitness models have provided useful unifying frameworks with which to capture such common trends found across these different scales and species, as they do not rely on system‐specific mechanistic details. In particular, Fisher's geometric model of adaptation (FGM), originally proposed simply as a convenient metaphor for phenotypic adaptation (Fisher, 1930), can correctly predict the oft‐observed shifted reflected Γ‐shape of the nearly neutral mode of the DFE (although not generally the strongly deleterious/lethal mode) (Bank et al., 2014; Bataillon & Bailey, 2014; Chevin, Martin, & Lenormand, 2010; Jacquier et al., 2013; Martin, 2014; Martin & Lenormand, 2006; Tenaillon, 2014; Trindade, Sousa, & Gordo, 2012). In addition, FGM predicts the common patterns of epistasis observed between beneficial mutations in systems of all scales (see also below): a general predominance of negative (antagonistic) epistasis and, more specifically, a trend of diminishing returns (Blanquart, Achaz, Bataillon, & Tenaillon, 2014; Martin, Elena, & Lenormand, 2007; Rokyta et al., 2011). These agreements are not so surprising when we consider the similarities of a Gaussian FGM to the sigmoidal and simple concave phenotype–fitness functions previously demonstrated to explain these patterns: in all cases, as the fitness maximum is approached, the upward slope becomes increasingly flat (*i.e*. they are all locally concave at high fitness). One difference is that, as opposed to the sigmoidal and simple concave functions, the nonmonotonicity of FGM also predicts sign epistasis (e.g. a mutation that is beneficial in a maladapted background may become deleterious in a well‐adapted background due to optimum overshooting (Blanquart et al., 2014)). Overall, the success of FGM suggests that at least some of the repeatedly observed properties of the genotype–fitness relationship may be remarkably predictable without the need for any mechanistic knowledge.

### 4.3 Genome‐wide epistasis (intergenic epistasis)

**Figure 10:**
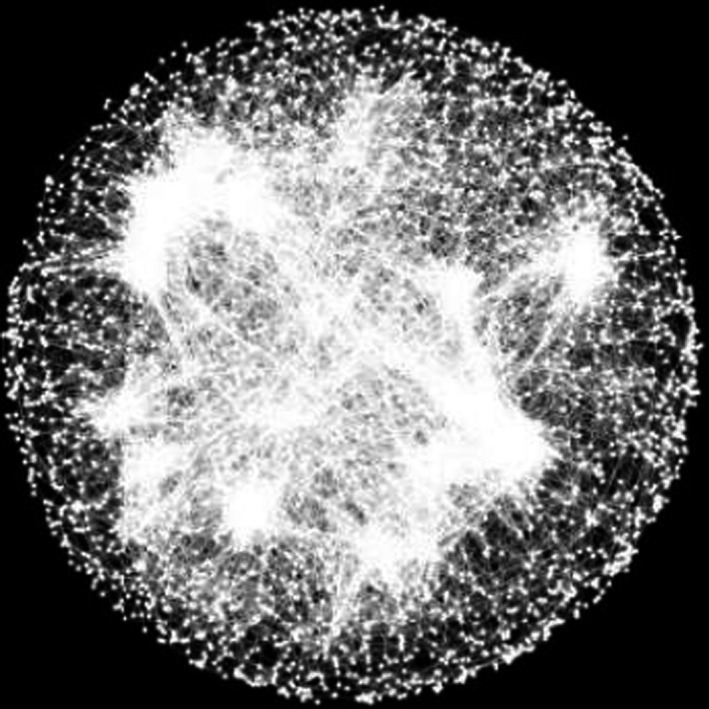
A genome‐wide network of gene–gene interaction profile similarities. Nodes are yeast genes, and edges connect genes with similar genome‐wide fitness interaction profiles, revealing functional modules. [From *Science*, *353*(6,306), aaf1420‐2, A global genetic interaction network maps a wiring diagram of cellular function (Costanzo et al., 2016). Reprinted with permission from AAAS**]**

The nature of epistasis appears to be less general across systems. Genome‐wide deletion analyses (like the single gene and regulatory sequence studies) tend to find a predominance of negative interactions, though still alongside a substantial proportion of positive ones (Babu et al., 2014; Costanzo et al., 2010, 2016; Onge et al., 2007; Roguev et al., 2008; Szappanos et al., 2011), but it should be noted that these have mainly been performed in yeast for now. In line with flux balance analysis (FBA) pairwise gene perturbation predictions (He, Qian, Wang, Li, & Zhang, 2010; Segrè, DeLuna, Church, & Kishony, 2005; Szappanos et al., 2011), comparing interaction profiles for different genes has provided information on the topology of their functional connections, providing “wiring diagrams” of cell function (Costanzo et al., 2016) (Figure 10). For example, genes from redundant pathways tend to undergo negative synergistic interactions with each other, and genes from the same pathway tend towards positive antagonistic interactions (perhaps largely responsible for the differential proportions of negative and positive epistasis) (Avery & Wasserman, 1992; Battle, Jonikas, Walter, Weissman, & Koller, 2010; Beltrao, Cagney, & Krogan, 2010; Breslow et al., 2008; Fu, Deng, Jin, Wang, & Yu, 2017; Lehner, 2011; Onge et al., 2007; Ye et al., 2005). As stated above, though, it is not clear that these rules should generalize to mutations other than the complete loss‐of‐function ones making up the vast majority of these data sets (hypomorphic alleles of essential genes are the exception) (Szathmary, 1993; Xu, Barker, & Gu, 2012).

On the other hand, epistasis between naturally occurring mutations in viral and cellular genomes appears to be biased towards positive interactions, with positive epistasis more common between the relatively frequent deleterious mutations and negative epistasis more common between rarely occurring beneficial mutations, that is an overall trend of antagonism (Bonhoeffer, 2004; Burch, 2004; Caudle et al., 2014; H.‐H. Chou et al., 2011; Chou et al., 2014; de Visser et al., 2011; Flynn et al., 2013; Khan et al., 2011; Kryazhimskiy et al., 2014; J Lalić & Elena, 2012; Maisnier‐Patin et al., 2005; Rokyta et al., 2011; Sanjuan, Moya, & Elena, 2004a; Schoustra, Hwang, Krug, & de Visser, 2016). This antagonism represents a kind of genomic buffering process: combinations of deleterious mutations are “less bad” than expected from simple additivity, and beneficial combinations are “less good”, but a mechanistic explanation is lacking. Beneficial/deleterious combinations have rarely been studied explicitly. Further, the trend of diminishing returns between beneficial mutations found in single genes is often also found at the genome scale, suggesting analogously the saturation, and sometimes even optimum overshoot (Rokyta et al., 2011), of some phenotype contributing to fitness (Berger & Postma, 2014). These general fitness‐level trends found for real mutations are rather encouraging for the predictability of adaptive dynamics, despite the underlying genetic and even phenotypic complexity (Kryazhimskiy et al., 2014).

## 5 THE ENVIRONMENT

It is clear that the environment affects genotype–phenotype relationships, and so for a complete understanding of them we must consider them across different environments. Indeed, the few studies that explore large mutant sets across large numbers of environments find fundamental changes, such as the proportion of essential genes (Nichols et al., 2011). Even such ambitious large‐scale studies explore only a vanishing fraction of potentially relevant fixed environments, though, not to mention dynamic ones.

Studies examining environmental effects therefore tend to have the ambition of proof of principle, rather than exhaustive sampling, and these have produced myriad examples of the environmental dependence of both mutation effects and epistasis. It remains extremely difficult to form any general conclusions, as “environment” may refer to the concentration of small molecules (gene expression inducers, enzyme substrates, cofactors, antibiotics) which have some specific role in the system of study (Dean, 1995; Lagator et al., 2016; Lagator, Paixão, et al., 2017; Melnikov et al., 2014; Nghe et al., 2018; Shultzaberger et al., 2010; de Vos, Dawid, Sunderlikova, & Tans, 2015; de Vos, Poelwijk, Battich, Ndika, & Tans, 2013; Wrenbeck et al., 2017), precise physicochemical parameters known to matter in in vitro studies (Hayden, Ferrada, & Wagner, 2011; Hayden & Wagner, 2012), or more general “pleiotropic” factors such as temperature, chemical stresses, complex nutrients or even host organism (Bank et al., 2014; Caudle et al., 2014; Dandage et al., 2018; Flynn et al., 2013; Fragata et al., 2018; Hietpas, Bank, Jensen, & Bolon, 2013; Jagdishchandra Joshi & Prasad, 2014; Lalić, Cuevas, & Elena, 2011; Li & Zhang, 2018; Mavor et al., 2016). It will be important going forward to develop a more systematic approach to the environment, focussing either on environments likely to provide mechanistic insight into mutation effects, or simply on those most relevant to industry or nature.

## 6 CONCLUDING REMARKS

Recent technological advances have now made it possible to score large‐scale genetic libraries for a variety of phenotypes in massively parallel fashion (“deep mutational scanning”) (Fowler & Fields, 2014; Hietpas et al., 2011). In the short term, the resulting genotype–phenotype maps have illuminated several trends such as the bimodality of mutational effects and the pervasiveness of epistasis, and allowed for rigorous testing of mechanistic biological models. In the long term, they open the possibility of developing predictive genetic models, which would launch a new era in bioengineering, precision medicine, infectious disease control and bioconservation, to name a few. Such quantitative models are limited by the precision of the large data sets informing them (Rubin et al., 2017). Great strides have been made here—while early studies were limited in numbers of replicates due to their expense, genetic barcoding strategies now enable each genotype to be represented by multiple independent lineages in a single experiment, allowing characterization of biological and experimental noise and even error correction (Fowler et al., 2014; Mavor et al., 2016). Further, a critical decision for any deep‐sequencing‐based assay is how best to make use of a defined number of sequencing reads (*e.g*. depth per replicate, number of replicates, number of time points/selection cycles), and computational analysis has revealed some general guidelines for maximizing experimental power (Matuszewski, Hildebrandt, Ghenu, Jensen, & Bank, 2016).

An exciting future direction is the large‐scale characterization of mutation effects at many phenotypic scales simultaneously (*e.g*. protein stability, protein activity, flux, expression, cell morphology, fitness), which could enable a direct and complete mechanistic description of the translation of genotype into high‐level traits (Cambray et al., 2018). Indeed, certain phenotypes, such as metabolic flux (Sauer, 2004) and the set of –omes, have received very little direct attention, mostly due to the enormous technical challenges and cost involved in their high‐throughput measurement/coupling to genotype libraries. One promising candidate, however, is the transcriptome, as it can be sequenced using RNA‐seq: using the same emulsion‐based technology that can enable distal genomic sites to be linked together for many single cells (Figure 9), the transcriptome can in principle be quantified for single cells while linking this information to the cells’ genotypes with the use of unique cellular DNA barcodes (Adamson et al., 2016; Dixit et al., 2016). The transcriptomic impact of random mutations or a large set of transcriptional regulator mutations, for example, could thus be rapidly assessed and even linked to high‐throughput fitness measurements.

Studies involving a few genes interacting at the level of elementary biological components (regulatory or metabolic motifs) can reveal surprisingly diverse types epistasis while allowing mechanistic interpretations of their origin. One promise is to integrate these local views to explain genome‐wide epistasis from biological mechanisms. Smaller‐scale studies may also allow the identification of generic principles which could be extrapolated to larger systems, for example the categories of phenotype to fitness relationships which generate certain types of epistasis or not. From a large‐scale evolutionary perspective, it is not currently clear whether upscaling leads to an increase in the complexity of interactions, or could rather lead to a form of averaging, as suggested theoretically (Martin, 2014).

Finally, the systematic analysis of the effects of genome‐wide combinations of point mutations still appears far out of reach, but a feasible next step might be introducing synthetic promoter libraries like those used in Keren et al. (2016) in front of *pairs* of genes across the genome and measuring the fitness effects. Although clearly artificial, and in some cases breaking regulatory links that are ensured by native promoters, such an experiment could provide quantitative two‐gene expression–fitness landscapes for many pairs of genes, which should be an extremely important component of the genotype–fitness relationship, and which for now we are almost completely blind to (see Martin, 2016 for higher level 2‐D trait–fitness landscapes in a multicellular organism).

With constantly improving technologies for reading and writing genotypes on a massive scale, and the application of experimental creativity, our mechanistic understanding of the genotype–phenotype relationship across different scales can only continue to flourish.

## Data Availability

Data sharing is not applicable to this article as no new data were created or analysed in this study.
